# Effects of moderate intensity endurance training vs. high intensity interval training on weight gain, cardiorespiratory capacity, and metabolic profile in postnatal overfed rats

**DOI:** 10.1186/s13098-018-0374-x

**Published:** 2018-09-26

**Authors:** Carlos Gabriel de Lade, Ana Eliza Andreazzi, Mariana Bolotari, Vinícius Moreira Gonçalves Costa, Vera Maria Peters, Martha de Oliveira Guerra

**Affiliations:** 1 Reproductive Biology Center, Federal University of Juiz de Fora, Rua José Lourenço Kelmer, s/n. Campus Universitário, Juiz de Fora, MG 36036-900 Brazil; 2 Department of Medicine, Federal University of Juiz de Fora, Juiz de Fora, MG Brazil

**Keywords:** Childhood obesity, Obesity management, Insulin resistance, Glucose intolerance, Aerobic exercise, High-intensity interval training, Rats

## Abstract

**Background:**

Obesity is associated with several comorbidities, such as cardiovascular disease and type 2 diabetes mellitus, and may have its origin in early life stages, such as in the lactation period, through metabolic programming. Physical activity aids in decreasing the chances of developing cardiovascular and metabolic diseases, even with small weight losses and, in children, can play an essential role in preventing weight gain and other health problems. The present study aimed to evaluate the effects of moderate intensity endurance training and high intensity interval training (HIIT) protocols on obesity-related parameters and cardiorespiratory capacity in overfed Wistar rats throughout the breastfeeding period.

**Methods:**

Two days after birth, forty male and female Wistar rats were clustered into two groups: Control Litter Group (CL; ten animals/litter) and Reduced Litter Group (RL; four animals/litter). At weaning, RL animals were distributed randomly into three experimental groups: sedentary, moderate intensity endurance training and HIIT, while CL animals were clustered into a sedentary group.

**Results:**

RL male and female body weight, before weaning, was significantly higher when compared with CL animals. This difference was maintained between CLSed and RLSed groups after weaning during all assessed periods. Adiposity was significantly higher in RLSed males when compared to CLSed males, and alterations in glycaemic metabolism were also observed. Endurance and HIIT protocols were efficient in improving maximal cardiorespiratory capacity, as well as concerning the glycemic metabolism and central fat accumulation of males and females submitted to childhood overfeeding by the litter reduction method.

**Conclusions:**

Both moderate endurance training and HIIT protocols included in early life were efficient in reverting or preventing certain metabolic alterations as a consequence of overfeeding during breastfeeding in male and female Wistar rats.

**Electronic supplementary material:**

The online version of this article (10.1186/s13098-018-0374-x) contains supplementary material, which is available to authorized users.

## Background

Post-natal development is considered an important event with respect to plasticity and maturation of physiological systems, which makes it more sensible to environmental influence. Furthermore, it may directly contribute to the development of long-term diseases, such as type 2 diabetes mellitus (DM2) and overweight, among others [1], a phenomenon known as metabolic programming [2].

In rodents, overfeeding throughout the breastfeeding period can be induced by litter reduction, leading to rises in caloric intake due to less competition for breastfeeding. Besides presenting an early hyperglycaemia profile, fat accumulation and overweight [3], rats and mice from reduced litters may present overweight, obesity, insulin resistance, hyperinsulinemia and hyperleptinemia, hyperphagia, glucose intolerance, dyslipidaemia and blood pressure rises in adult life [4].

In humans, evidence demonstrates that 150–250 min of moderate physical activity per week, with a calorie burn of around 1.200–2.000 kcal, would be enough to prevent weight gain. In addition, physical activity aids in decreasing the chance of cardiovascular and metabolic disease development, improves physical fitness and health indicators in obese individuals, even with small weight losses [5, 6] and, in children, can play an essential role in preventing weight gain and other health problems [7].

Sustained sessions of moderated aerobic exercises or endurance, performed along several weeks, increase the oxidative capacity of skeletal muscles, modifying the energy source used during effort episodes, resulting in increased aerobic capacity. However, recent studies demonstrate that High Intensity Interval Training (HIIT) alters the energetic metabolism of skeletal muscles, and is similar to certain adaptations caused by a moderate aerobic training [8, 9].

An increased body of evidence in healthy individuals has demonstrated that HIIT brings cardiovascular and metabolic benefits, similar to or greater than those reached by moderate endurance training [10–13]. However, reports found in literature still indicate some controversy with respect to the comparison between HIIT protocol efficacy and moderate endurance protocols related to strategies focused on the management of overweight/obesity and related problems. In addition, the relation between the regular practice of different types of physical training, including during childhood, in individuals overfed during the breastfeeding period requires further study. In this context, the present study aimed to evaluate the effects of moderate intensity endurance training and high intensity interval training protocols on obesity-related parameters and cardiorespiratory capacity on overfed Wistar rats throughout breastfeeding period.

## Methods

### Animals

Twenty-four pregnant Wistar rats were used throughout this study, obtained from the Reproductive Biology Center’s (CBR) breeding facilities at the Federal University of Juiz de Fora (UFJF), whose offspring, forty males and forty females, were used in the experiment. Two days after birth, the animals were clustered into two groups: Control Litter Group (CL; ten animals/litter) and Reduced Litter Group (RL; four animals/litter) in order to compose, the experimental groups after weaning. Litter reduction was accomplished 2 days after birth, according to Bei et al. [4], and animal management aiming to arrange litters composed only by males or females followed the same author.

The animals were maintained in ventilated ALESCO^®^ cabinets with controlled air flow under standard laboratory conditions, with controlled humidity, ventilation, temperature of 22 ± 2 °C and a 12:12-h light–dark cycle. After weaning (21 days after birth) the animals were fed standard feed NUVILAB CR1^®^ pellets (Nuvital Nutrientes Ltda. Colombo, Brazil) and water ad libitum. All procedures were approved by the UFJF Ethics Committee on Animal Use (CEUA/UFJF), Protocol No. 045/2016.

### Experimental groups

At weaning, RL animals were distributed randomly into three experimental groups: sedentary (RLSed), endurance (RLEnd) and HIIT (RLHIIT), while CL animals comprised a sedentary group (CLSed). Additional file 1: Figure S1 illustrates the flow chart used as guide for the configuration of the experimental groups.

### Cardiorespiratory fitness tests (VO_2_ max) and training protocols

Immediately after weaning, animals were submitted to an adaptation period on a motorized treadmill with 6 stands (Insight^®^) for 5 days of running, during 10 min and progressive speed, where they were trained afterwards.

Before, during and after the training protocols, all animals, including those belonging to the sedentary groups, were evaluated concerning maximum aerobic capacity (VO_2_ max), on a metabolic treadmill (Panlab^®^) by using a gas analyser (Harvard Aparatus^®^). The protocol involved running on a treadmill until exhaustion, at a 5° inclination, starting at a 6 m/min speed, increasing speed at 3 m/min every 3 min until animals were unable to continue [14].

Apart from the assessment criteria for cardiorespiratory fitness and training efficiency, the results from the tests performed after 4 and 8 weeks of training were used for a better adjustment of the training load, making it more individual and according to the aerobic capacity of each animal.

After forming the experimental groups, two physical training protocols were used, namely endurance and HIIT. The endurance protocol was characterized by a moderate intensity (65–70% VO_2_ max), 60 min per session, for 8 weeks. Each training session consisted of 10 min as a warm-up (50% VO_2_ max) and 50 min for the main routine (65–70% of VO_2_ max). The adjustment of the training load was delivered during the 4th week of training, after a new VO_2_ max assessment. HIIT sessions encompassed about 40 min, split into 10 min as a warm-up (50% VO_2_ max) and six 3-minute periods running at high intensity (85–90% VO_2_ max) alternated with 2-min periods running at low intensity (50% VO_2_ max), summing 30 min for the main routine. All training protocols began after an adaptation period and the first VO_2_ max test, which was carried out at about the 30th post-natal day, and were performed 3 times per week on alternate days.

### Oral glucose tolerance test (OGTT) and Insulin tolerance test (ITT)

For both tests, animals were submitted to a 6-h fasting period. Blood was collected through a lancet in the animal’s tail so as to gauge capillary glycaemia by using an AccuChek Advanced (Roche^®^, Germany) glucometer. The tests were carried out a week before euthanasia, at 72-h intervals.

During the OGTT test, basal glycaemia (T0) was measured and, subsequently, 2 g/kg of body weight of a 50% glucose solution was administered via force feeding. Soon after, blood samples were collected at times T1 (15′), T2 (30′), T3 (60′) and T4 (120′). The total area under the OGTT curve was calculated.

During ITT, the animals received an intraperitoneal infusion of insulin (1 U/kg of body weight) and blood samples were collected before (T0′) administering the insulin and at times T1 (5′), T2 (15′), T3 (30′) and T4 (45′) after the insulin administration. The constant rate for glucose disappearance (K_iit_) was computed through the formula 0.693/(t1/2), where t1/2 is calculated from the slope of the smallest quadratic analysis of plasma glucose concentrations after insulin administration [15].

### Body weight, visceral adipose and food intake

CL and RL body weight were monitored weekly from the fourth post-natal day on, until the 90th day of life. The Lee index (LI), which assesses overweight/obesity, was computed through the formula [LI = (BM × 1/3)/NAL × 100], where BM = body weight (g); NAL = nasoanal length (NAL) (cm). Food intake was also registered weekly by computing the difference between the amount of offered and remaining food, 24 h after delivery.

Animals were euthanized via diaphragmatic rupture after all “in vivo” procedures were carried out. The anaesthetic protocol combined 90 mg/kg (Vetanarcol, Konig, Brasil) of the dissociative anaesthetic Ketamine hydrochloride and 10 mg/kg (Kensol, Konig, Brasil) of the muscle relaxant of xylazine hydrochloride. Both drugs were blended and administered together via the intraperitoneal route. After blood extraction through cardiac puncture under general anaesthesia, the collected blood was centrifuged, and the serum was stored at − 80 °C. Immediately after euthanasia, retroperitoneal and perigonadal adipose tissues were removed and weighed on a fine scale (Bioprecisa^®^-specificity 0.0001 g—Brasil).

### Biochemical serum analysis

The biochemical analysis was carried out on an automatized CobaS c111 (Roche^®^) device using Cobas c111 kits for total cholesterol HDL, LDL, triglycerides and blood glucose.

### Statistical analyses

First, the Shapiro–Wilk test was applied in order to verify data normality. Aiming to detect possible alterations in RL animals, comparisons between CLSed groups and their respective RLSed groups were carried out by applying a t-Student or Mann–Whitney test. Regarding possible alterations between sex and types of training, a two-way ANOVA was applied, while a one-way ANOVA, followed by a Tuckey post hoc test was applied when comparing, separately, RL males and females from the same group. In order to obtain the OGTT results, the total area under the glycaemia curve was computed. Results are expressed as mean ± SD (standard deviation) at a significance level of p < 0.05. All statistical analyses were carried out using the SPSS (SPSS Inc^®^, versão 21) and GraphPad Prism^®^ 5.0 (San Diego, USA) softwares.

## Results

RL males and females body weight, before weaning, was significantly greater from the fourth and 10th day of life on, respectively, when compared with CL animals. This difference remained steady between CLSed and RLSed groups after weaning during all assessed periods (Additional file 2: Figures S2A and S2B). No significant differences between RLSed, RLEnd and RLHIIT male body weight after weaning until the 90th day of life were detected (Additional file 2: Figure S2C). However, for females, this difference was registered only in the last measurement point, where CLSed presented a statistically higher body weight mean compared to RLEnd and RLHIIT (Additional file 2: Figure S2D).

RLSed males presented significantly higher food intake for eight out of nine measurements assessed with respect to CLSed (Additional file 2: Figure S2E). Regarding CLSed and RLSed females, differences in food intake were only detected for five out of nine measurement points, where RLSed reached higher values, (Additional file 2: Figure S2F). As for the food intake of RLSed, RLEnd and RLHIIT animals, significant differences were registered among males during the 2nd week assessment, where RLSed presented a higher mean consumption compared to RLEnd and RLHIIT (Additional file 2: Figure S2E). In females, significant differences were observed during the 7th week, where RLSed and RLEnd presented higher mean consumption compared to RLHIIT (Additional file 2: Figure S2F).

Additional file 3: Figures S3A and S3B display the comparisons between maximum oxygen consumption at days 30 (VO_2_-1), 60 (VO_2_-2), 90 (VO_2_-3) for the CLSed and RLSed groups of both males and females.

No statistically significant differences in any of the three assessed time periods were observed for males and females from the CLSed and RLSed groups. Nevertheless, when comparing the VO_2_ max test results between the first (VO_2_-1) and the third (VO_2_-3) test, a depletion oxygen consumption for the CLSed and RLSed groups, both males and females was observed. In males, the difference between VO_2_-1 and VO_2_-3 reached 19% (CLSed) and 30% (RLSed), while in females, the difference reached 19% (CLSed and RLSed).

With respect to the comparison among RL animals, groups trained in endurance and HIIT showed statistically significant differences between the third test results (VO_2_-3) and the first test results (VO_2_-1), both for males and females, pointing out the efficiency of both training protocols concerning increased breathing capacity in trained animals. In males, the differences reached 29% in the RLEnd group and 21% in the RLHIIT group. The same behaviour was observed for trained females, with 24% and 18% increases in the RLEnd and RLHIIT groups, respectively. However, concerning the sedentary groups, 30% and 19% drops in the VO_2_ max in the RLSed groups of both males and females, respectively, were observed.

No statistically significant differences between the means overfed groups of males and females trained in endurance and HIIT protocols were observed for the last VO_2_ max results. Nevertheless, the RLEnd group of males presented VO_2_ max 61% higher than the values reached by the RLSed group, while the difference between the RLHIIT and RLSed groups reached 59%. The same behaviour was observed for females, where the RLEnd and RLHIIT groups reached 47% and 39% higher values than those presented by the RLSed group respectively.

OGTT results for males and females of the CLSed, RLSed, RLEnd and RLHIIT groups are displayed in Additional file 4: Figures S4A and S4C. The area under the curve in RLSed males was 11.2% higher when compared to CLSed animals statistically significant. This difference was also observed for OGTT female results, in which the RLSed group exhibited a 9% higher area under the curve 9 in comparison with the CLSed group. OGTT results for RL animals showed no statistically significant differences, neither for males or female of endurance and HIIT trained groups when compared to the RLSed groups (Additional file 4: Figures S4B and S4D).

Concerning the Insulin Tolerance Test (ITT), RLSed males showed a constant rate of glucose disappearance means (K_iit_), 39% lower when compared to the CLSed group in the ITT, statistically significant, demonstrating major resistance to insulin in the overfed group (Additional file 4: Figure S4E). No difference concerning the K_iit_ means between the CLSed and RLSed groups for females was observed (Additional file 4: Figure S4F).

Among RL males (Additional file 4: Figue S4E), the RLSed group reached lower values of constant rate of glucose disappearance (K_iit_) throughout the ITT when compared to both the RLEnd group (51%) and the RLHIIT group (60%), demonstrating major resistance to insulin in sedentary overfed animals when compared to endurance and HIIT trained groups. However, among females, the RLHIIT group reached a higher mean K_iit_ than animals from the RLSed (23%) and RLEnd (30%) groups. In other words, HIIT training was more efficient in increasing sensitivity to insulin in females compared to endurance training (Additional file 4: Figure S4F).

Table 1 displays body weight 90 days after birth, nasoanal length, the Lee index and adiposity (relative weights of perigonadal and retroperitoneal adipose tissues) of males and females belonging to the CLSed, RLSed, RLEnd and RLHIIT groups. Both RLSed males and females reached significantly greater body weight values when compared to their respective CLSed groups. On the other hand, no differences were observed for the NAL and the Lee index. Adiposity, represented by the relative weights of perigonadal and retroperitoneal adipose tissues, was significantly higher in RLSed males when compared to CLSed males, at 26% and 58%, respectively.

**Table 1:** Body mass, Lee Index, NAL and adiposity of the male and female groups at the 90th day

	Males			
	CLSed	RLSed	RLEnd	RLHIIT
BM (g)	272.70 ± 22.27	303.25 ± 26.75^a^	297.6 ± 24.42	295.15 ± 38.37
NAL (cm)	23.60 ± 0.52	24.12 ± 0.74	24.05 ± 0.63	24.00 ± 0.96
Lee Index	27.51 ± 0.55	27.84 ± 0.63	27.68 ± 0.28	27.76 ± 0.59
Adiposity (%)				
Perigonadal	1.55 ± 0.30	1.87 ± 0.31^a^	1.56 ± 0.18*	1.57 ± 0.35^#^
Retroperitoneal	1.50 ± 0.48	2.25 ± 0.50^a^	1.45 ± 0.30*	1.62 ± 0.47^#^

	Females			
	CLSed	RLSed	RLEnd	RLHIIT
BM (g)	176.45 ± 10.59	196.5 ± 17.72^b^	174.95 ± 15.84*	173.85 ± 16.73^#^
NAL (cm)	20.5 ± 0.6	21.34 ± 0.74	21.12 ± 0.23	20.98 ± 0.46
Lee Index	26.11 ± 0.36	26.76 ± 0.81	26.11 ± 0.20	26.01 ± 0.21
Adiposity (%)				
Perigonadal	1.43 ± 0.37	2.08 ± 0.58^b^	1.82 ± 0.41	1.38 ± 0.43^#^
Retroperitoneal	1.38 ± 0.63	1.81 ± 0.44	1.35 ± 0.35*	1.51 ± 0.41

Results are presented as means and standard deviations. A total of 10 rats were used from both groups for all parameters

*BM* body mass, *NAL* nasoanal length

*^,#^Significant difference compared to RLSed (p < 0.05)

^a, b^Significant difference compared to CLSed (p < 0.05)

A significant difference regarding relative retroperitoneal adipose tissue weight was observed for females, where the RLSed group reached a means 45% higher in comparison to the CLSed group. Despite not presenting a significant difference, the RLSed group means was 31% higher compared to the CLSed group means.

No statistically significant differences for final body weight, NAL, or the Lee Index were observed between RL male groups, although differences were noted for adiposity. The RLSed group presented higher means for relative perigonadal adipose tissue weight in relation to RLEnd (20%) and RLHIIT (19%) and also for the relative retroperitoneal adipose tissue weight in relation to the two trained groups, with 38% (RLEnd) and 32% (RLHIIT) differences, respectively. RLSed females reached a statistically higher final body weight means than RLEnd (12%) and RLHIIT (13%) females. In addition to body weight differences, RLSed presented a statistically higher relative perigonadal tissue weight compared to the RLHIIT group (51%) and a statistically higher relative retroperitoneal tissue weight compared to the RLEnd group (34%). No NAL and Lee Index differences between groups of RL females were observed.

A significant difference as observed for male CLSed and RLSed biochemical serology, namely serum glucose, where RLSed registered a 14% higher means. Despite no statistical differences, the means of total cholesterol and triglycerides in the RLSed groups were 36% and 10% higher when compared to the CLSed means, respectively. A significant difference for female HDL was also observed, higher in the CLSed group compared to the RLSed group (Table 2).

**Table 2:** Biochemical variables of the male and female groups at the 90th day

	Males			
	CLSed	RLSed	RLEnd	RLHIIT
Blood glucose (mg/dL)	168.97 ± 26.76	192.83 ± 12.32^a^	169.57 ± 26.22^*^	163.82 ± 17.59^#^
Total cholesterol (mg/dL)	63.8 ± 10.63	70.09 ± 11.69	67.95 ± 8.95	70.84 ± 8.56
HDL (mg/dL)	56.53 ± 8.93	61.50 ± 9.53	61.48 ± 7.96	63.00 ± 7.05
LDL (mg/dL)	9.86 ± 1.66	9.83 ± 1.42	10.41 ± 2.88	9.55 ± 2.57
Triglycerides (mg/dL)	30.91 ± 10.09	42.07 ± 13.83	33.66 ± 15.80	36.81 ± 11.55

	Females			
	CLSed	RLSed	RLEnd	RLHIIT
Blood glucose (mg/dL)	168.59 ± 15.33	166.06 ± 13.15	149.22 ± 21.55^*^	153.92 ± 7.09
Total cholesterol (mg/dL)	70.82 ± 4.80	71.54 ± 6.18	67.28 ± 11.78	62.22 ± 7.67
HDL (mg/dL)	64.77 ± 6.41	56.88 ± 5.86^b^	60.73 ± 10.83	61.95 ± 4.22
LDL (mg/dL)	7.70 ± 1.50	8.79 ± 2.68	7.24 ± 1.58	6.30 ± 1.51^#^
Triglycerides (mg/dL)	26.18 ± 4.82	30.40 ± 5.51	27.67 ± 5.62	25.47 ± 2.27

Results are presented as means and standard deviations. A total of 10 rats were used from both groups for all parameters

*^, #^Significant difference compared to NRSed (p < 0.05)

^a, b^Significant difference compared to CLSed (p < 0.05)

Serum glucose means in RLSed males groups were 14% and 18% higher than in males from the RLEnd and RLHIIT groups, respectively. Among females, the RLSed group registered a statistically higher glucose serum means compared to the RLEnd group (11%). Despite no statistical difference, the RLSed group registered an 8% higher means than the RLHIIT group. In addition, LDL means in the RLSed group was significantly higher than the RLHIIT group (39%) and 21% higher that the RLEnd mean, albeit non-significantly.

Furthermore, no interactions between sex and type of training were observed for any of the assessed variables.

## Discussion

Metabolic syndrome prevalence has increased, mainly worsened by infant obesity rises, which, in turn, lead to increased health problems risks in overweight children [16]. As obesity tends to be maintained during adulthood, it is reasonable to think of this condition as maintained during adulthood in overweight children [17, 18]. The overweight/early obesity induction protocol via litter reduction is currently widely applied, as it allows for the imitation of overfeeding in humans, common when using nutritive formulas, for instance, which may imply in metabolic alterations in adult life [4].

Evidence classifies physical exercises and regular physical activity as a non-medicine treatment option for the control and prevention of children and adult obesity, with a noticeable improvement in the metabolic condition, even with body weight alterations [19]. Nevertheless, children are not usually involved in appropriate physical activity programs, as only one out of three children can be considered physically active. Increasing regular physical activity practice is considered as a crucial intervention on children’s health [7, 19].

In the present study, RLSed animals presented higher body weight than their respective CLSed since the 1st days of life until the 90th day after birth, also consuming higher food amounts. However, displaying more relevance compared to the observed differences in body weight, both male and female RLSed animals registered a relative weight gain in perigonadal and retroperitoneal adipose tissues. Some years ago, the litter reduction model was applied in order to demonstrate that not only was somatic rat growth affected, but a disproportional increase of body fat deposition was also detected [20]. The results of the present study corroborate those reported by Habbout et al. [21] since, according to the authors, overfeeding during breastfeeding has been related to a higher probability of hyperphagia, overweight and obesity and, in rodents, tends to maintain after weaning, even when offspring is fed standard feed.

RLSed males presented alterations in their glycemic metabolism compared to CLSed males, displaying higher serum glycaemia, reduced glucose tolerance (OGTT) and a superior resistance to insulin, expressed as a lower K_iit_ value. Regarding females, the RLSed group presented reduced glucose tolerance (OGTT) only. Several studies have focused on the effects of early overfeeding and alterations in the insulin-glucose axis in adult animals, since these animals frequently exhibit augmented glycaemia and insulin values, as well as unbalances in the insulin-glucose axis [22, 23]. Overfeeding during breastfeeding may lead to modifications of insulin signalization routes in skeletal muscles, increasing visceral fat accumulation and insulin resistance [24], in agreement to the results reported herein.

According to the reported results, the glycemic metabolism of RLSed males was more damaged by overfeeding during breastfeeding when compared to RLSed females. This difference between males and females corroborate a claim made by Basset and Graig [25], who demonstrated higher sensitivity to insulin in female adipocytes, due to an increase in receptor connectivity and, consequently, higher cellular glucose uptake.

The present study aimed to evaluate different physical exercises programs integrated in the early life of animals overfed during breastfeeding. When compared to the RLSed group, both RLEnd and RLHIIT males and females registered gains in VO_2_ max at the end of 8 weeks training, with significant differences when compared to the RLSed groups, but with no differences between them, demonstrating the efficiency of both training protocols to reach VO_2_ max. Fisher et al. [26] distributed 28 overweight men into HIIT training and moderate endurance groups, for 6 weeks, concluding that both were effective and with no differences concerning cardiorespiratory fitness gain. Kong et al. [27] also verified that both training types were efficient at increasing VO_2_ max in young and obese women. This data is relevant clinically, since VO_2_ max gain is associated to minor risks for cardiometabolic disease appearance and death for all reasons [7, 28].

In comparison to their respective RLSed, the trained groups presented lower relative adipose tissue weight, with no differences between both training types concerning central fat gain. Clinically, this data becomes crucial, since lower fat volume located in the abdominal cavity is directly related to minor risks for cardiometabolic disease development [29]. Türk et al. [30], concluded that HIIT was more effective at decreasing body fat in comparison with endurance training in obese adults. On the other hand, Kong et al. [27] verified that obese endurance trained women presented significant improvement in body composition, that did not occur in the HIIT trained group. Ficher et al. [26] concluded that both HIIT and endurance were effective and presented no significant differences regarding body fat ratio reduction in overweight men.

Both training protocols were effective at improving the glycemic metabolism of trained RL animals, with a drop in glycaemia levels during fasting in endurance and HIIT trained males and females, despite non-significant differences with RLHIIT females. Clinically, increases in impaired fasting glucose in infants have been associated with increased DM2 risk in adulthood [31] and the results reported herein show equivalence of both types of exercises beginning in the childhood period on hyperglycaemia prevention in adult RL animals. On the other hand, no OGTT differences for both males and females belonging to the RLEnd and RLHIIT groups when compared to the CLSed group were detected, concluding that both adopted protocols were inefficient at improving insulin tolerance in trained RL animals. In spite of this, RLEnd and RLHIIT males presented higher sensitivity to insulin in the ITT, as well as RLHIIT females, when compared to their respective RLSed groups.

The glycemic metabolism of RL males is more impacted by overfeeding during breastfeeding compared to RL females [25], which may explain the absence of differences concerning insulin sensitivity between CLSed and RLSed females. Some studies indicate some similarity between HIIT and endurance effects on insulin sensitivity [26], while others report that higher intensity HIIT training may be more decisive in improving this parameter, even among healthy individuals [32], which can explain the higher sensitivity to insulin observed in RLHITT females.

Moderate endurance exercise is usually prescribed aiming to improve body composition, physical capacity and certain health parameters in children and adults. However, interest in HIIT protocols has increased recently, mainly due to the same benefits brought by moderate endurance training, but less lengthy. Despite increased studies comparing both types of exercises, only some compare their effects on overweight children. Araújo et al. [33] compared moderate endurance and HIIT protocol effects in overweight children, concluding that both types of training were efficient at improving peak VO_2_ and general health condition. Two meta-analyses [32, 34] compared HIIT protocols and moderate endurance. The authors observed that HIIT can be considered more effective in improving several health parameters, such as VO_2_ max and insulin resistance, with a lower training volume. However, the same authors point out certain constraints, such as the amount of included studies and intervention duration, which requires further research to reach more robust conclusions.

The present study is relevant since, in contrast to experimental studies involving metabolic alterations and physical exercise, animals were submitted to training protocols at an early stage, specifically, soon after weaning. Results indicate that the adopted moderate endurance and HIIT protocols were efficient at improving some conditions caused by overfeeding during breastfeeding, mainly glycemic metabolism parameters and prevention of central fat gain. Regular physical exercise is beneficial in early life, even for inadequately fed individuals during a crucial development stage and, consequently, at higher risk for metabolic alterations. These results could aid in physical exercises program composition, as part of non-medical treatment aiming at losing weight and obtaining metabolic improvement in early overweight children, as well as reducing risks for cardiometabolic diseases in adult life. Further studies involving litter reduction model and different physical exercise protocols, such as type, duration, intensity and weekly frequency, are required in order to better understand the influence of physical activity in individuals with early body weight alterations and possible long term metabolic alterations.

## Conclusions

Both moderate intensity endurance training and HIIT protocols included in early life were efficient concerning cardiorespiratory capacity gain, lower relative adipose tissue weight, and improvement in glycemic metabolism, reverting or preventing metabolic alterations as consequence of overfeeding during breastfeeding, in both male and female Wistar rats.

## Additional files

**Additional file 1: Figure S1.** Study flow chart. RL: reduced litter group; CL: control litter group; HIIT: high intensity interval training.

**Additional file 2: Figure S2.** Male and female body mass and food intake. Male (A) and female (B) body mass before weaning. CLSed, RLSed, RLEnd and RLHIIT male (C) and female (D) body mass after weaning. CLSed, RLSed, RLEnd and RLHIIT male (E) and female (F) food intake after weaning. Results are presented as means and standard deviation. Ten rats were used for both groups for all parameters. ^*^CL and RL intergroup differences; ^#£¥^RL intragroup differences. p < 0.05.

**Additional file 3: Figure S3.** Male (A) and female (B) maximal oxygen uptake (VO_2_ max). Results are presented as means and standard deviation. Ten rats were used in both groups for all parameters. ^*#£¥^intragroup differences at VO_2_ max-1 and VO_2_ max-3; ^ab^differences between NREnd/NRHIIT groups compared to NRSed at VO_2_ max-3. p < 0.05.

**Additional file 4: Figure S4.** Oral glucose tolerance test (OGTT) and Insulin tolerance test (ITT) in males and females. Male OGTT (A) and (B) respective area under the curve; Female (C) OGTT and (D) respective area under the curve; (E) Male ITT and respective K_itt_; (F) Female ITT females and respective K_itt_. ^ab*^Same letters or symbols: significant differences. p < 0.05.

## Abbreviations

CL: control litter
BM: body mass
DM: diabetes *mellitus*
DM 2: type 2 diabetes *mellitus*
END: endurance training
HDL: high-density lipoprotein cholesterol
HIIT: high intensity interval training
ITT: insulin tolerance test
LDL: low-density lipoprotein cholesterol
NAL: nasoanal length
OGTT: oral glucose tolerance test
RL: reduced litter
VO_2_ max: maximal oxygen uptake
