# Supplementary figures and images for: Effects of moderate intensity endurance training vs. high intensity interval training on weight gain, cardiorespiratory capacity, and metabolic profile in postnatal overfed rats
Source: Diabetol Metab Syndr. 2018 Sep 26;10:70. doi: 10.1186/s13098-018-0374-x (PMC6158819; doi:10.1186/s13098-018-0374-x)

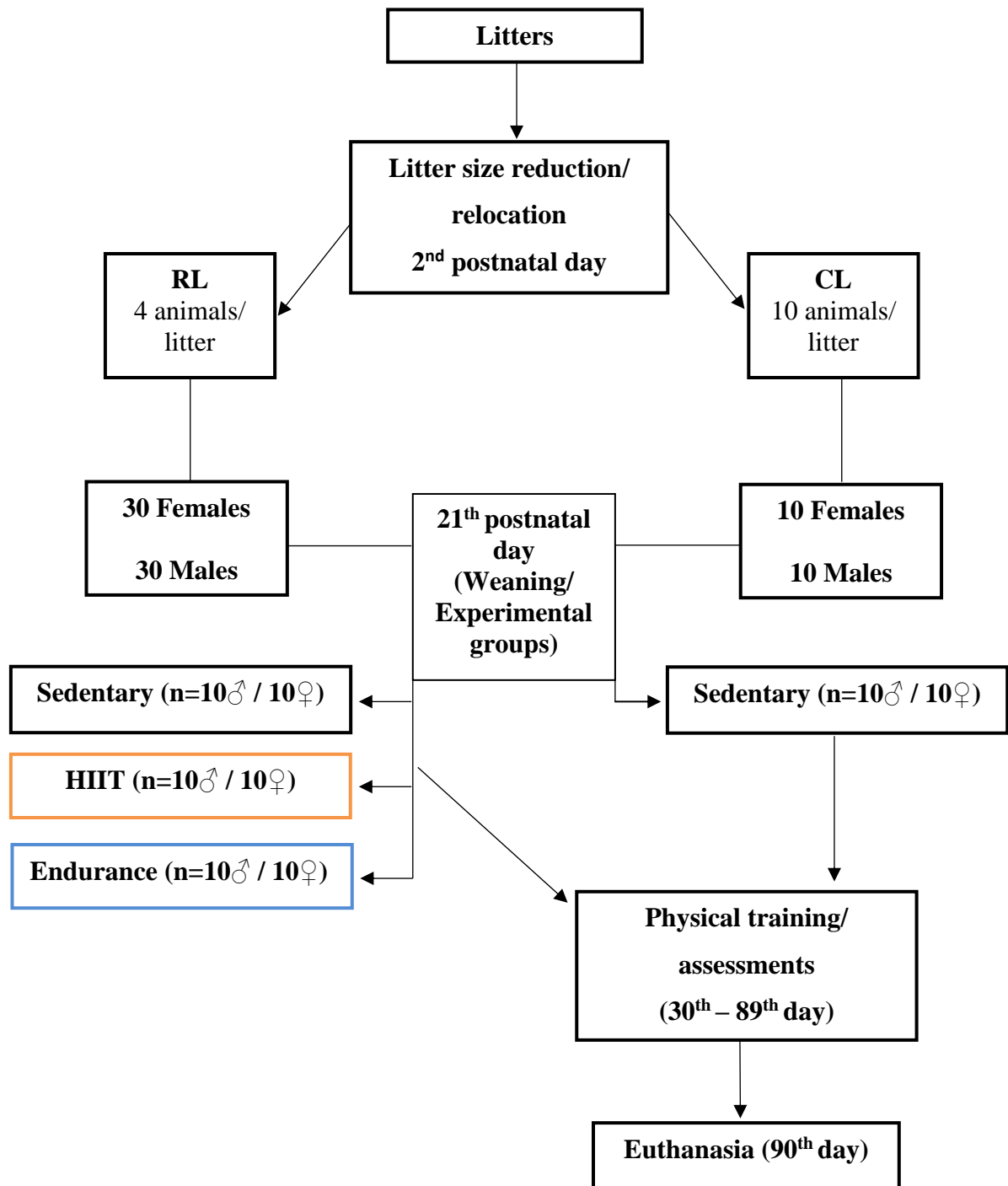

Supplement: Supplementary file 1 — Additional file 1: Figure S1. Study flow chart. RL: reduced litter group; CL: control litter group; HIIT: high intensity interval training. [file 13098_2018_374_MOESM1_ESM.pdf]

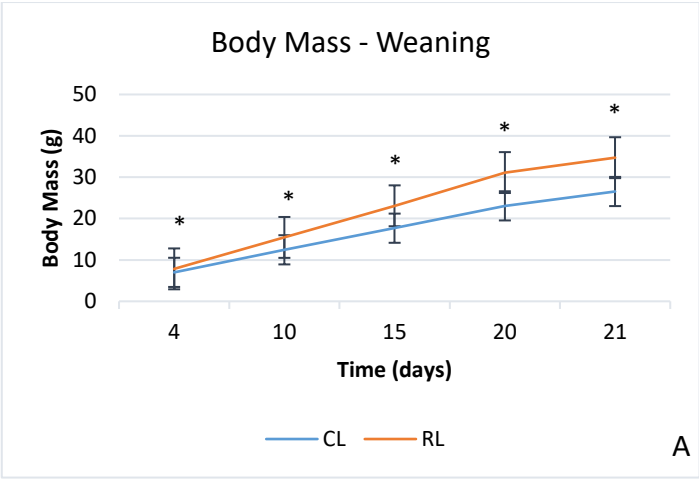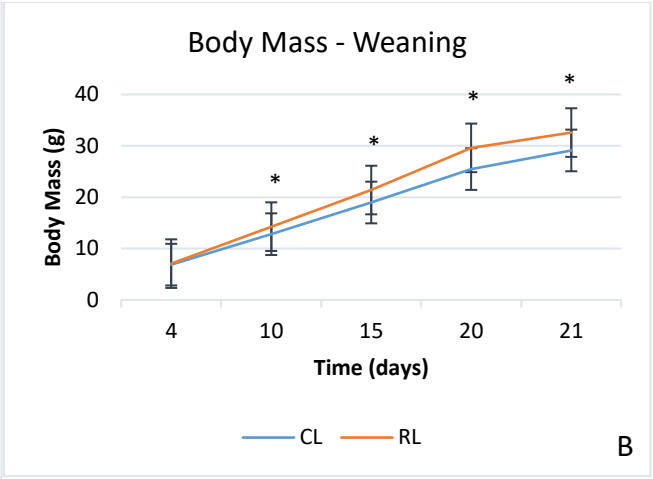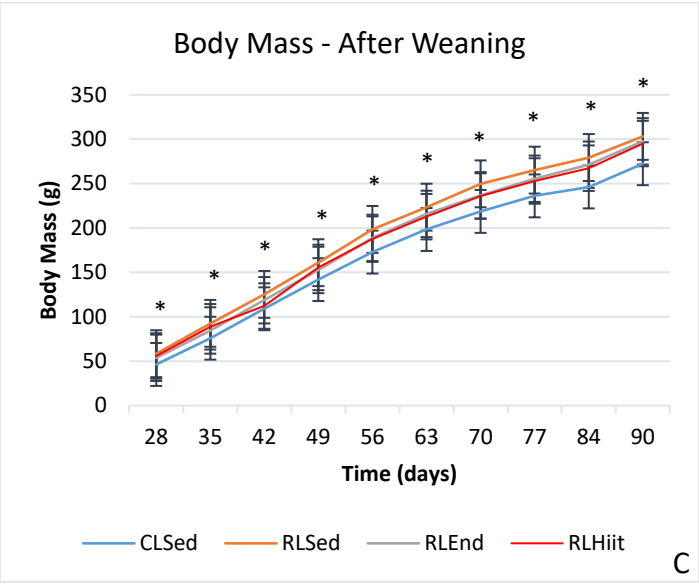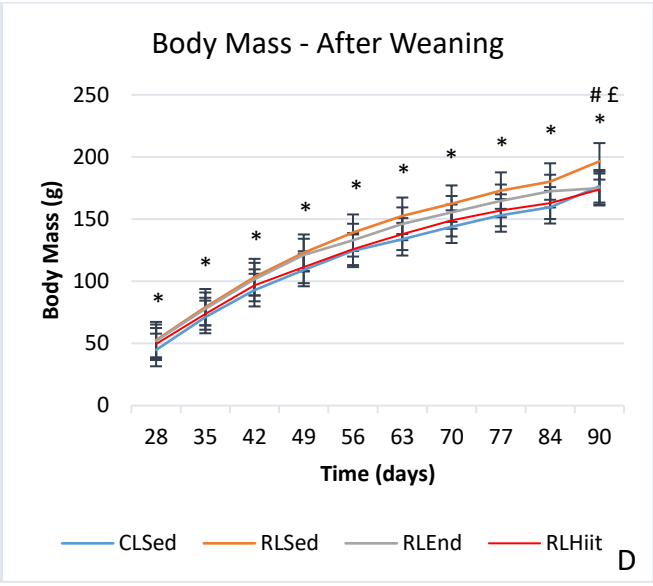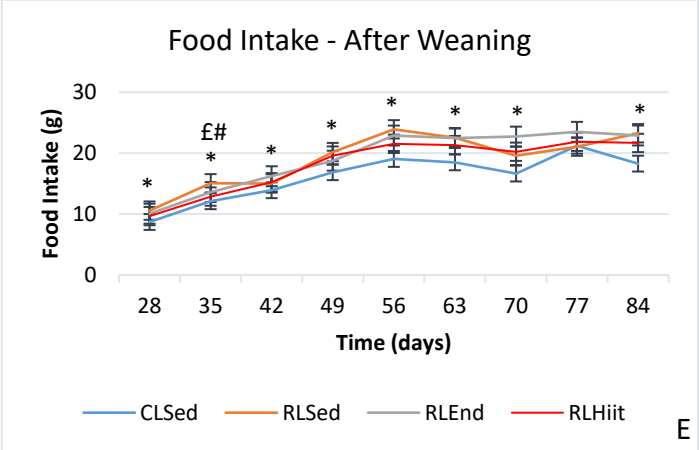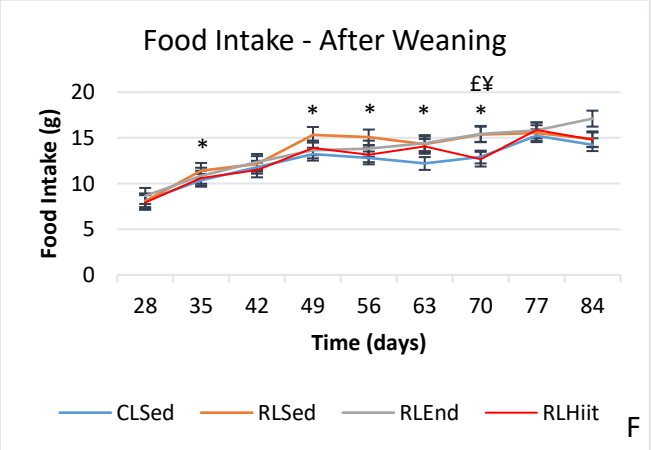

Supplement: Supplementary file 2 — Additional file 2: Figure S2. Male and female body mass and food intake. Male (A) and female (B) body mass before weaning. CLSed, RLSed, RLEnd and RLHIIT male (C) and female (D) body mass after weaning. CLSed, RLSed, RLEnd and RLHIIT male (E) and female (F) food intake after weaning. Results are presented as means and standard deviation. Ten rats were used for both groups for all parameters. *CL and RL intergroup differences; #£¥RL intragroup differences. p < 0.05. [file 13098_2018_374_MOESM2_ESM.pdf]

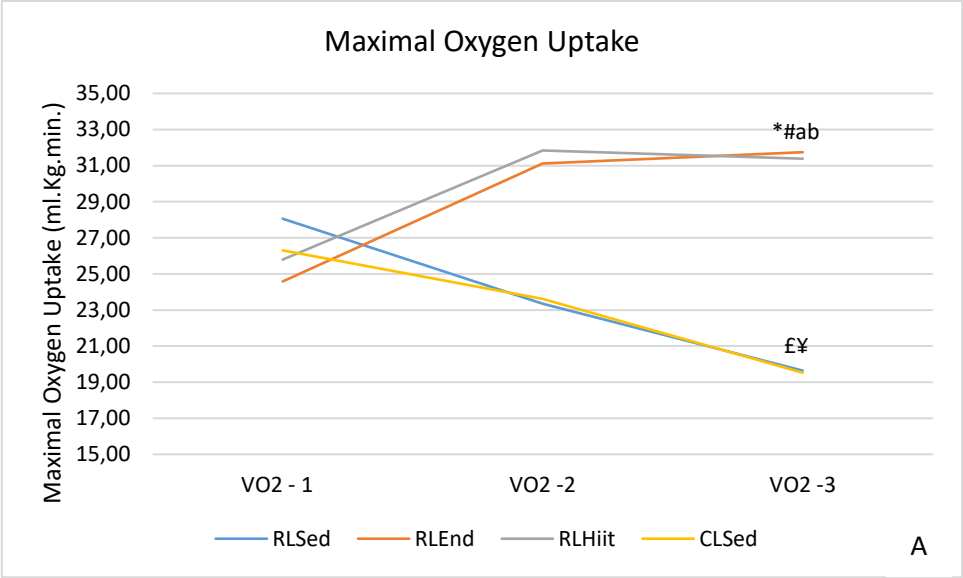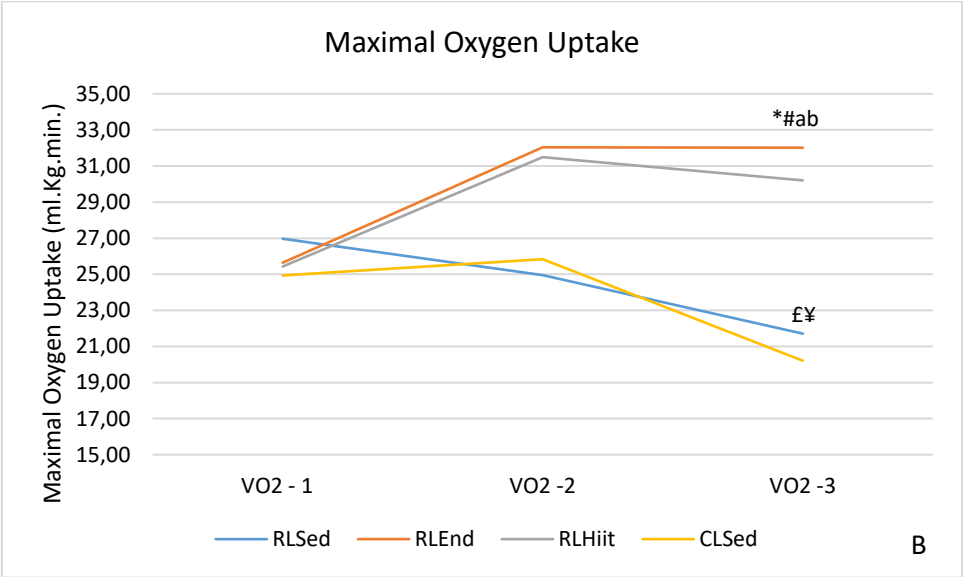

Supplement: Supplementary file 3 — Additional file 3: Figure S3. Male (A) and female (B) maximal oxygen uptake (VO2 max). Results are presented as means and standard deviation. Ten rats were used in both groups for all parameters. *#£¥intragroup differences at VO2 max-1 and VO2 max-3; abdifferences between NREnd/NRHIIT groups compared to NRSed at VO2 max-3. p < 0.05. [file 13098_2018_374_MOESM3_ESM.pdf]

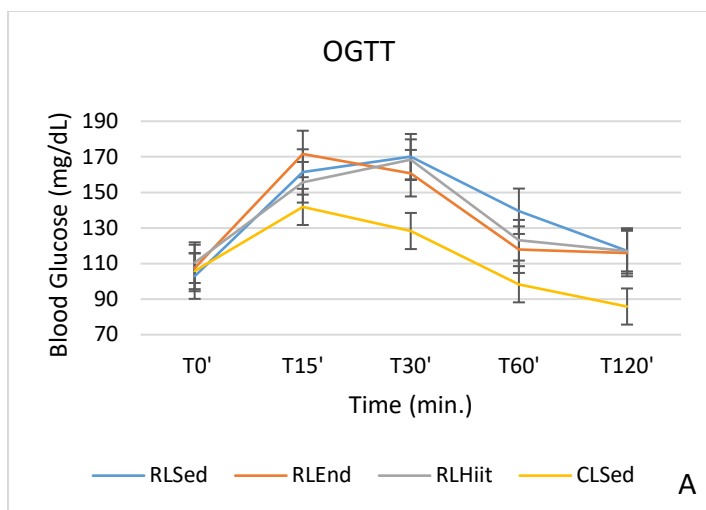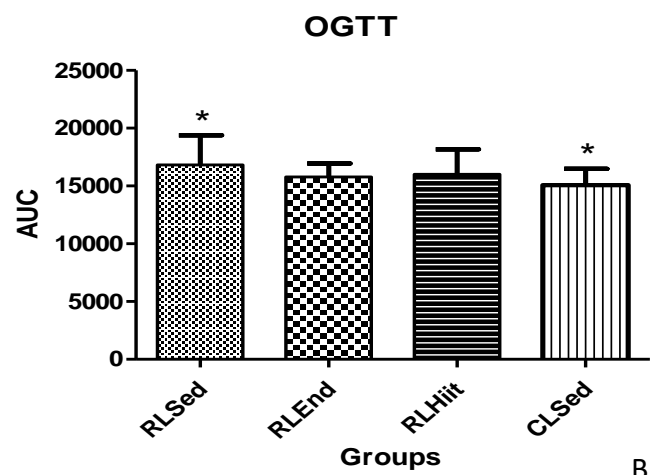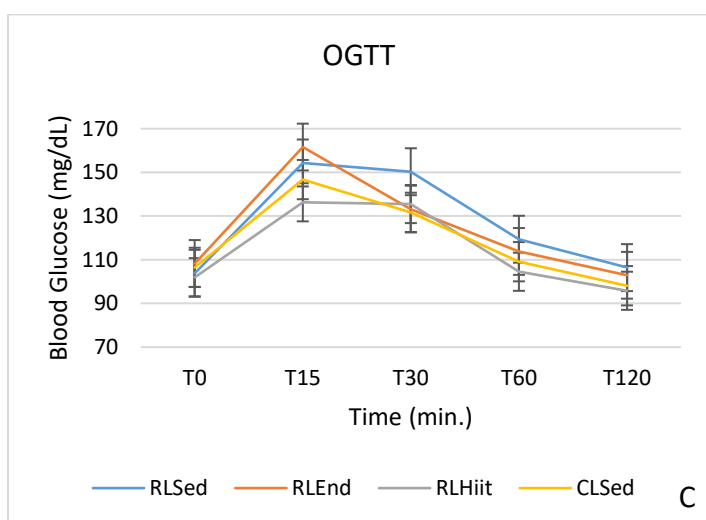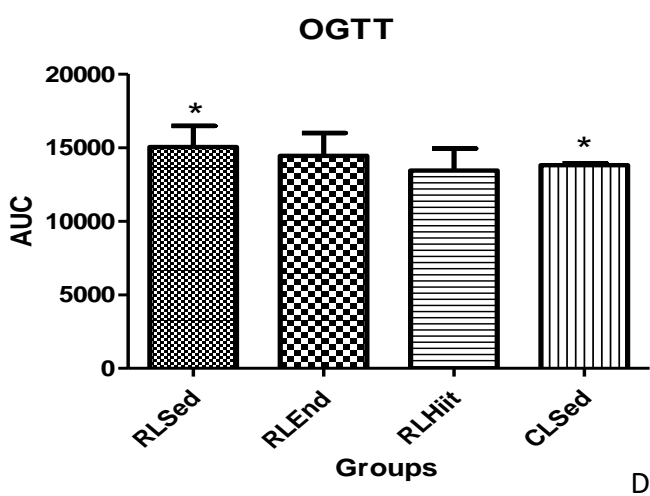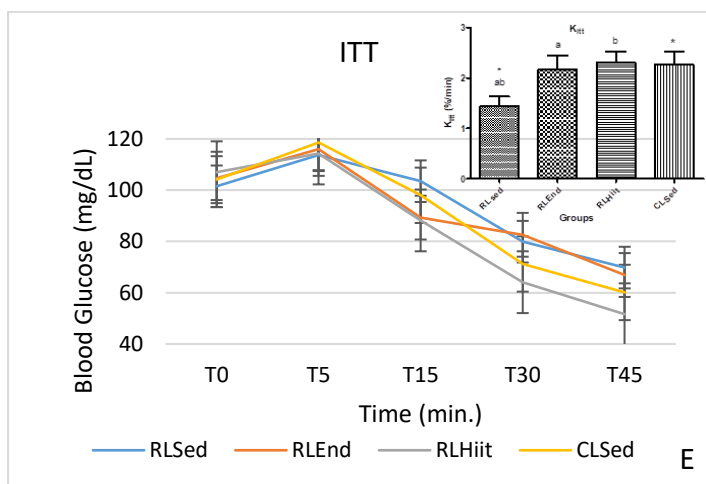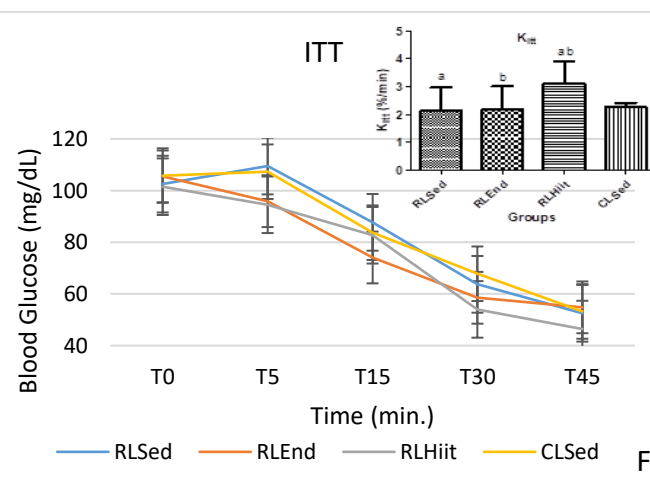

Supplement: Supplementary file 4 — Additional file 4: Figure S4. Oral glucose tolerance test (OGTT) and Insulin tolerance test (ITT) in males and females. Male OGTT (A) and (B) respective area under the curve; Female (C) OGTT and (D) respective area under the curve; (E) Male ITT and respective Kitt; (F) Female ITT females and respective Kitt. ab*Same letters or symbols: significant differences. p < 0.05. [file 13098_2018_374_MOESM4_ESM.pdf]
